# Modulation of the inwardly rectifying potassium channel Kir4.1 by the pro-invasive miR-5096 in glioblastoma cells

**DOI:** 10.18632/oncotarget.16949

**Published:** 2017-04-07

**Authors:** Dominique Thuringer, Gaetan Chanteloup, Jonathan Boucher, Nicolas Pernet, Christophe Boudesco, Gaetan Jego, Aurelien Chatelier, Patrick Bois, Jessica Gobbo, Laurent Cronier, Eric Solary, Carmen Garrido

**Affiliations:** ^1^ INSERM, U1231, Faculty of Medicine, 21000 Dijon, France; ^2^ CNRS ERL 7368, STIM Laboratory, 86022 Poitiers, France; ^3^ University of Bourgogne-Franche-Comté, 21000 Dijon, France; ^4^ CGFL, Department of Medical Oncology, 21000 Dijon, France; ^5^ INSERM, U1170, Institut Gustave Roussy, 94508 Villejuif, France

**Keywords:** K^+^ current, exosome, migration, microRNA, filopodia

## Abstract

Inwardly rectifying potassium channels (Kir), and especially the barium-sensitive Kir4.1 encoded by *KCNJ10*, are key regulators of glial functions. A lower expression or mislocation of Kir4.1 is detected in human brain tumors. MicroRNAs participate in the regulation of ionic channels and associated neurologic disorders. Here, we analyze effects of miR-5096 on the Kir4.1 expression and function in two glioblastoma cell lines, U87 and U251. Using whole-cell patch-clamp and western-blot analysis, we show that cell loading with miR-5096 decreases the Kir4.1 protein level and associated K^+^ current. Cell treatment with barium, a Kir4.1 blocker, or cell loading of miR-5096 both increase the outgrowth of filopodia in glioma cells, as observed by time-lapse microscopy. Knocking-down Kir4.1 expression by *si*RNA transfection similarly increased both filopodia formation and invasiveness of glioma cells as observed in Boyden chamber assay. MiR-5096 also promotes the release of extracellular vesicles by which it increases its own transfer to surrounding cells, in a Kir4.1-dependent manner in U251 but not in U87. Altogether, our results validate Kir4.1 as a miR-5096 target to promote invasion of glioblastoma cells. Our data highlight the complexity of microRNA effects and the role of K^+^ channels in cancer.

## INTRODUCTION

Under physiological conditions, one of the main functions of glial cells is to maintain ionic homeostasis in the brain during neuronal activity. This function is achieved through a process called potassium spatial buffering, which involves a specific subset of potassium (K^+^) selective ion channels known as inwardly rectifying K^+^ (or Kir or IRK) channels (for review, see [1]). Among the Kir channel family, Kir4.1, which is encoded by *KCNJ10* gene, is predominantly expressed in glial cells of the brain. This channel determines neuronal excitability and axonal conduction through maintaining a high K^+^ permeability of the plasma membrane and a negative resting membrane potential in glial cells (for review, see [2]). There are growing evidence implicating Kir4.1 in various neurological diseases, *i.e*. mutation in *KCNJ10* gene causes an autosomal recessive disorder associating early onset seizures, ataxia, mental retardation, and electrolyte imbalance [3] while a polymorphism in this gene correlates with seizure susceptibility [4]. The Kir4.1 dysfunction is also involved in developmental disorders including autism, in the disruption of ionic homeostasis that follows central nervous system trauma, ischemic events and inflammation, epilepsy and in neurodegenerative diseases in which it could open therapeutic perspectives [5].

A reduced expression or mislocation of Kir4.1 channels was detected in human brain tumors including low- and high-grade astrocytomas and oligodendrogliomas [6–9]. The consecutive lack of Kir current compromises the buffering capacity of glial cells, leading to cytotoxic edema. Because miR-5096 was specifically detected in glioma cells [10] and bioinformatics tools (BLASTN, https://blast.ncbi.nlm.nih.gov) identified a potential target site for miR-5096 on *KCNJ10* gene (overlap, 91%), we examined the impact of this microRNA on Kir4.1 expression and functions in U87 cells, a human glioblastoma cell line derived from a human astrocytoma of grade IV [11], and the U251 glioblastoma (grade III-IV) model [12–15]. We show that miR-5096 could specifically inhibit Kir4.1 in glioma cells. This microRNA also increased their extracellular vesicle release and filopodia outgrowth. Because we and others have shown that miR-5096 could be transferred from glioblastoma cells to astrocytes [10] and to human microvascular endothelial cells (HMEC) [16] through heterocellular gap junctions, we also examined its effects on HMEC.

## RESULTS

### miR-5096 inhibits the barium-sensitive current by targeting Kir4.1 in U87 glioma cells

**Figure 1:**
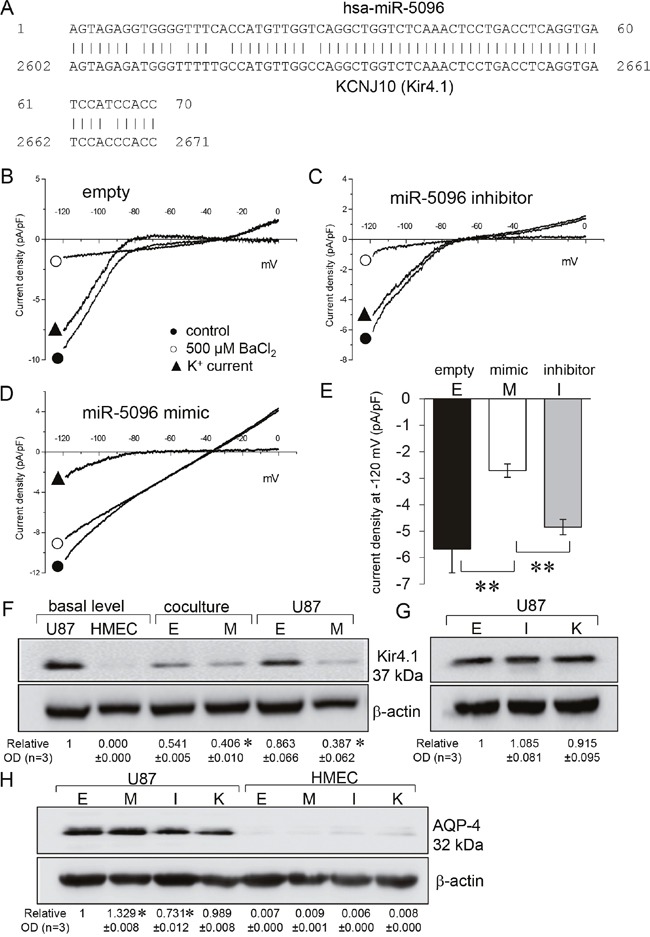
miR-5096 inhibits Kir4.1 current **(A)** Graph showing the *KCNJ10* gene contained a matched binding site of miR-5096 (overlap, 91%). **(B-D)** Whole-cell recordings showing barium-sensitive currents in glioblastoma cells transfected empty **(B)**, miR-5096 inhibitor (30 nM; **C**) and miR5096 mimic (30 nM; **D**). After 48h of cell culture, currents were recorded by applying voltage ramps from -120 to 0 mV (holding potential of –60 mV), before and after external BaCl_2_ application (500 μM). **(E)** Mean current density of the barium-sensitive current recorded at -120 mV (mean ± SD; **P<0.01; n=6). **(F)** Immunoblot analysis of Kir4.1 protein in whole cell lysates from homotypic cultures and co-cultures of transfected U87 and HMEC. Whole cell lysates after 48 h of culture in FCS-free conditions. One representative of 3 independent experiments is shown (β-actin as loading control). Numbers indicated mean values of the mean ratio (±SD) of Kir4.1 protein related to β-actin optical densities (OD) normalized to the basal ratio observed in untreated cells (mean relative values ± SD; *P<0.05 *vs* empty; n = 3). Cells were loaded (M) or not (empty, E) with miR-5096 mimic (30 nM). **(G)** Effects of inhibitors on the expression of Kir4.1 protein. U87 cells were loaded (I) or not (empty, E) with miR-5096 inhibitor (30 nM), or exposed (K) to 500 μM BaCl_2_. Numbers indicated mean values of OD (normalized with β-actin) of bands relative to Empty (± SD; *P<0.05 *vs* empty; n = 3). **(H)** Immunoblot analysis of AQP-4 protein in whole cell lysates from homotypic cultures of transfected U87 and HMEC. Numbers indicated mean of OD (normalized with β-actin) of bands relative to Empty from different experiments (mean ± SD; *P<0.05 *vs* empty; n = 3).

The analysis and prediction from bioinformatics tools identified a potential targeting site for miR-5096 on *KCNJ10* gene (Figure 1A). The effects of miR-5096 on Kir current in U87 glioblastoma cells were first recorded in a whole-cell configuration by applying voltage ramps from -130 to 0 mV to eliminate transient voltage-dependent components. Specifically, we defined the Kir4.1 current as the current component inhibited by external barium (500 μM BaCl_2_), a well-known blocker of Kir4.1 channels [1, 17, 18]. Representative recordings (Figure 1B-1D) and the mean Kir current densities measured at -120 mV (Figure 1E) are shown. U87 cells transfected with an empty vector showed a typical inwardly K^+^ rectification that was inhibited by barium (Figure 1B, 1E). Similar K^+^ current traces and density could be recorded in cells loaded with a miR-5096 inhibitor (30 nM, Figure 1C, 1E). In contrast, the inward rectification was suppressed and the Kir current density was decreased by 50% in cells loaded with a miR-5096 mimic (30 nM; Figure 1D, 1E). Note the depolarization of the reverse potential of the current (≈ -40 mV).

Since Kir4.1 is the main inwardly rectifying K^+^ channel in glia cells [1, 19], we investigated the effects of miR-5096 on the expression of this protein. As expected, Kir4.1 was detected in U87 but not in HMEC (Figure 1F). Its expression was not significantly modified by co-culture of glioma cells with HMEC (ratio 1:1), nor by the transfection procedure. Cell loading with miR-5096 mimic dramatically decreased Kir4.1 expression in U87 (Figure 1F) while their loading with miR-5096 inhibitor and their exposure to barium, had limited effect if any on Kir4.1 expression (Figure 1G).

Kir4.1 channel was shown to be closely associated with the water channel protein aquaporin-4 (AQP-4) in the glial cell endfeet [7, 20, 21]. Therefore, we checked if miR-5096 also affected AQP-4 expression. AQP-4 was detected in U87, not in HMEC (Figure 1H). The miR-5096 inhibitor slightly decreased, while miR-5096 mimic slightly increased, AQP-4 expression in glioma cells (Figure 1H). Barium did not induce any significant change in the protein expression. Altogether, miR-5096 appeared to inversely regulate the expression of Kir4.1 and AQP-4.

### miR-5096 increases the release of extracellular vesicles and HSP90α by U87 glioma cells

**Figure 2:**
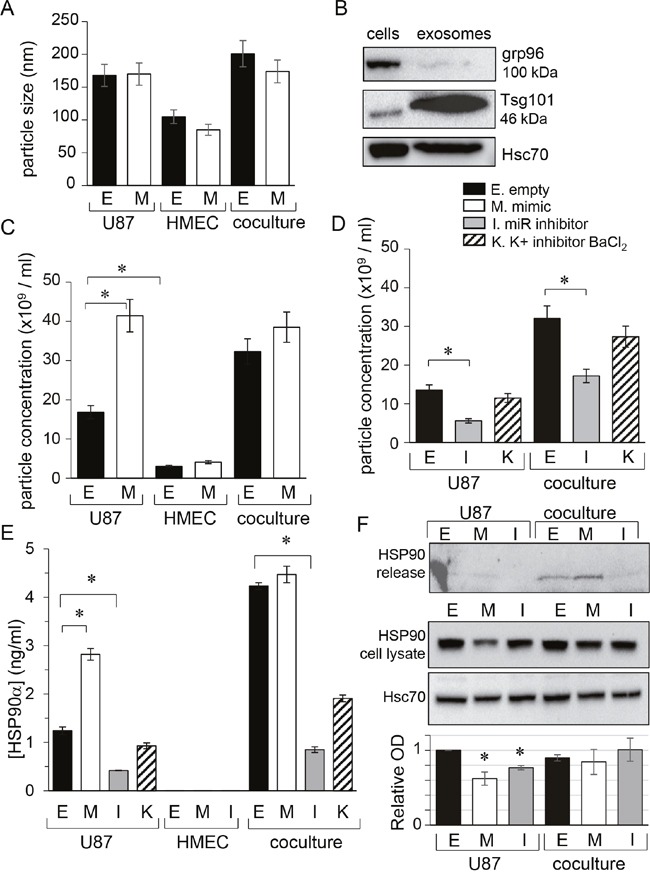
miR-5096 increases the release of exosomes from glioblastoma cells U87 and HMEC were transfected empty (E) or miR-5096 mimic (M; 30 nM) or miR5096 inhibitor (I; 30 nM) before culture and co-culture (ratio 1:1). When indicated, 500 μM BaCl_2_ was added to media of empty-transfected cells to block Kir currents (K). After 48h of culture, cell supernatants were collected. Exosomes were isolated by serial centrifugation protocol and characterized using Nanosight technology. **(A)** Mean size of exosomes isolated from homotypic cultures and co-cultures for 48 h. Transfections did not affect the size of exosomes (mean ± SD; P>0.05 *vs* empty for each group; *n* = 3). **(B)** Exosome validation by the presence of specific markers. Representative western blot for the exosome related protein Tsg101 and for the endoplasmic reticulum protein grp96 (63 μg proteins loaded per lane in 8% gel; Hsc70 as loading control). **(C, D)** miR-5096 (M) increased the concentration of exosomes released by U87 while inhibiting endogenous miR-5096 (I) decreased it. Blocking Kir current (K; 500 μM BaCl_2_) had no effect (mean ± SD; P>0.05 *vs* empty for each group; *n* = 3). **(E)** miR-5096 increased extracellular HSP90α released by U87 cells and not by HMEC. HSP90α was measured by ELISA in the soluble phase of supernatants from cultures and co-cultures (mean ± SEM; *P<0.05 *vs* empty for each group; *n* = 3). This is a dosage from the whole cell bath whereas the secretion by U87 must be much higher in the closed vicinity of neighboring cells. **(F)** miR-5096 decreased the cell expression of HSP90 in U87 cell cultures. The upper blot was performed with the cell supernatants from the same cultures shown in the lower blot. Whole cell lysates after 48 h of culture in FCS-free conditions. One representative of 3 independent experiments is shown (Hsc70 as loading control). Histogram indicated mean values of the optical density (OD; normalized with Hsc70) of bands from different experiments (± SD; *P<0.05 *vs* basal; n = 3).

Because cell membrane depolarization promotes the secretion of both neurotransmitters and cell-derived vesicles [22], we next examined the contribution of Kir4.1 current to the release of exosomes in our culture conditions. We collected supernatants of homotypic and heterotypic (ratio 1:1) cultures of HMEC and U87 cells for 48h then separated extracellular vesicles from soluble fraction using established protocols [23]. Characterization of extracellular vesicles was carried out by using Nanosight technology, showing sizes ranging from 85 to 150 nm (Figure 2A), and using immunoblotting to detect the expression of the exosome-related protein Tsg101 (Figure 2B). The endoplasmic reticulum protein grp96, not detected in extracellular vesicles, was used as a negative control (Figure 2B). Since intercellular transmission of microRNAs could occur without gap-junctions, we tested whether exosomes contained miR-5096 (see Supplementary Figure 1). For that purpose, we loaded U87 cells with miR-5096 mimic and collected exosomes after 48h in culture before using qPCR to detect the miR-5096 mimic in these exosomes. While the size of exosomes was not affected by the transfection procedure (Figure 2A), their number in mimic-transfected U87 cultures was twice that measured in empty-transfected cultures (from 18×10^9^ to 42×10^9^ exosomes/ml; Figure 2C). This number was not further increased by co-culture with HMECs and the number of exosomes remained low in HMEC cultures. Figure 2C also shows that supernatant of empty-transfected co-cultures (50% U87 plus 50% HMEC) contained more exosomes than homotypic U87 cultures (100% U87), suggesting that the presence of HMEC could promote the secretion of exosomes. The studied glioma cells produced more exosomes than microvascular cells and this production was increased by miR-5096 whereas miR-5096 specific inhibitor had no effect in exosome secretion (Figure 2D). Altogether, miR-5096 may behave as a promoter of glioma cell secretory functions through increasing the release of exosomes. We also examined extracellular HSP90α, a well-studied secreted chaperon [24] used as a marker of bad prognostic in glioblastoma [25]. Importantly, miR-5096 also increased twice the release of HSP90α by U87 cells but not by HMECs (Figure 2E). Of note, the secretion of HSP90α by U87 glioma cells was also amplified in the presence of HMEC and was decreased by loading the cells with miR-5096 inhibitor. However, the expression of HSP90 proteins in U87 cells did not reflect these extracellular HSP90 variations (Figure 2F). Cell loading with miR-5096 significantly decreased the cell content of HSP90 protein compared to empty-transfected cell cultures (Figure 2F). Finally, HSP90α secretion was not inhibited by external barium (500 μM BaCl_2_; Figure 2E), which was rather unexpected as membrane depolarization could favor cell secretion [26] but could be related to cell death.

### miR-5096 decreases *KCNJ10* gene expression in U87 and U251 glioblastoma cells

**Figure 3:**
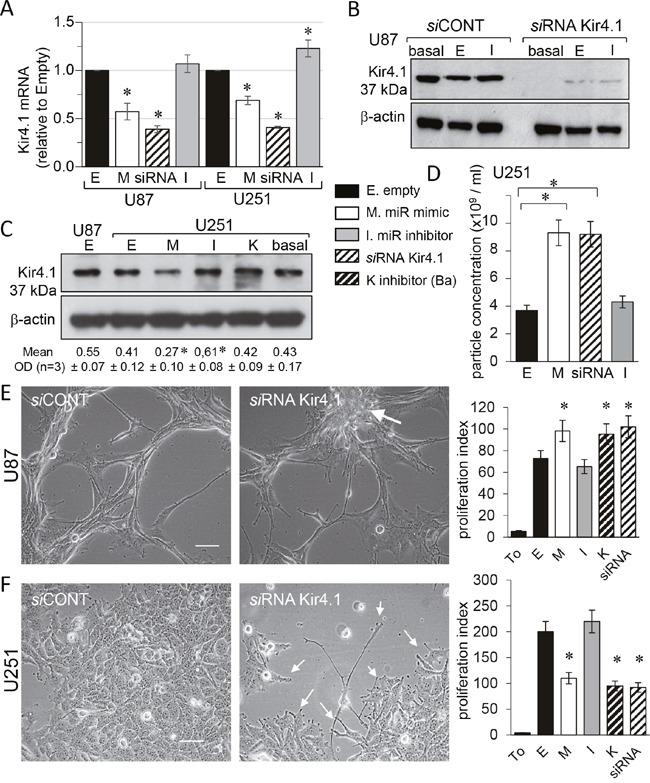
miR-5096 decreases KCNJ10 gene expression in U87 and U251 glioblastoma cells Homotypic cell cultures were loaded or not (empty, E; or basal level) with miR-5096 mimic (M) or inhibitor (I; 30 nM), transfected by Kir4.1 *si*RNA (siRNA) or exposed to 500 μM BaCl_2_ (K). **(A)** Cell loading miR-5096 decreased Kir4.1 mRNA expression in both U87 and U251 cells. Data are normalized values to GAPDH, expressed relative to the levels in Empty conditions and are mean ±SD (each measurement was made in triplicate; n=4; *P-values<0.05 *vs* empty). **(B)** Representative western blot of Kir4.1 protein level in U87 cells transfected with control or Kir4.1 *si*RNA for 2 days (n=5; Hsc70 as loading control). **(C)** Immunoblot analysis of Kir4.1 protein in whole cell lysates from U251 cells. One representative of 3 independent experiments is shown (β-actin as loading control). Numbers indicated mean values of relative optical density (OD) of bands (± SD; *P<0.05 *vs* basal; n = 3). **(D)** Loading miR-5096 mimic (M) or silencing Kir4.1 (siRNA), both increased the release of exosomes by U251 (mean ± SD; P>0.05 *vs* empty for each group; *n* = 3). **(E)** Kir4.1 siRNA transfection increased the proliferation of U87, as did miR5096 mimic. Images of cells showing finger-like projections (filopodia) and the cell growth in a “rosary” induced by silencing Kir4.1 expression (white arrow; scale bar, 50 μm). Right histogram: 48 h after transfection, cells were tested for additional 24 h of culture for tracking proliferation (T_0_, initial time point of experiment; mean ± SD; *P<0.05 *vs* empty; *n* = 3). **(F)** Kir4.1 siRNA transfection decreased the proliferation of U251 as did miR5096 mimic. Same protocols as in **(E)** While U251 cells proliferate in cobblestone monolayer in control conditions, they slow down their growth and emit finger-like projections (filopodia; white arrows) by silencing Kir4.1 expression.

In order to validate the observed effects, we have tested the responsiveness of the commonly used glioblastoma model U251 cells to miR-5096-loading. The gene expression level of Kir4.1 was firstly measured in the two glioma cell lines by using real-time RT-PCR. As shown in Figure 3A, cell loading with miR-5096 mimic resulted in a significant decrease in the expression of Kir4.1 at the mean by 43% and 31% in U87 and U251, respectively. In contrast, the miR-5096 inhibitor increased by 20% the expression of Kir4.1 gene only in U251 cells. Successful knockdown of Kir4.1 was observed using *si*RNA as seen by the reduced levels of protein (Figure 3B).

As reported by others [12], U251 cells expressed lower levels of endogenous Kir4.1 proteins than U87 (Figure 3C). Nevertheless, miR-5096 exerted an inhibitory effect on the Kir4.1 expression. Inhibition of endogenous miR-5096 produced a small but significant increase in its expression. As reported for U87 cells, the Nanosight analysis of cell supernatants revealed that U251 cells secreted exosomes of size between 90 to 130 nm and whose secretion in mimic-transfected cultures was twice that measured in empty-transfected cultures (Figure 3D). Such an increase was also observed by knocking down Kir4.1 (i.e. with *si*RNA). However, the observation of cell morphology revealed marked differences between the two cell lines (Figure 3E, 3F). The U87 cells typically showed finger-like projections (filopodia) and an accelerated cell growth in a “rosary” induced by silencing Kir4.1 expression (Figure 3E). In contrast, U251 cells which typically proliferated in cobblestone monolayer, slowed down their growth and emitted finger-like projections (filopodia) by silencing Kir4.1 expression (Figure 3F).

Because inhibition of Kir4.1 currents could modulate cell proliferation [27], we checked the impact of miR-5096 mimic, cell exposure to barium and Kir4.1 knockdown, on glioma cell number. A slight but significant increase in cell proliferation was observed 48 h after transfection in U87 (Figure 3E), whereas a two-fold decrease was in U251 cells (Figure 3F). Clearly Kir4.1 knockdown differently affected proliferation of the two glioma cell lines, suggesting that additional mechanisms are involved [12].

### miR-5096 and barium similarly increase filopodia outgrowth of glioma cells

**Figure 4:**
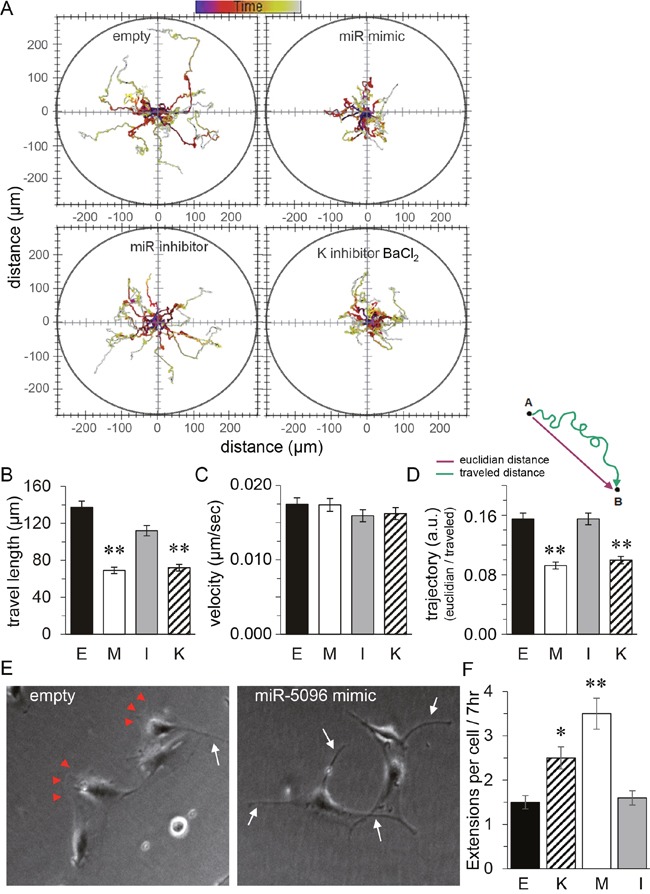
Similar effects of miR-5096 and barium on glioblastoma cell displacement 48h after transfection (E. empty; M. miR mimic; I. miR inhibitor), cells were plated in the absence or presence of 500 μM BaCl_2_ (K), then were imaged every 10 min for 24h. **(A)** Representative tracks of cell centroids migrating into the space (n=41). **(B)** Mean cell travel length (mean SD; **P<0.01, n=41). **(C)** Mean instantaneous cell velocity as in B. **(D)** Mean trajectories (arbitrary unit) characterize the motion of transfected U87; i.e. the persistence of cell to migrate to the initial direction (reflecting changes in the angle of migration). **(E)** Images of individual cells showing the wound edge (lamellipodia; arrow heads) and finger-like projections (filopodia; white arrows). Same experiment as in **(A)** but observed 7 h after re-plating. **(F)** Quantification of the mean number of filopodial extensions > 2μm in width in transfected cells plated without serum for 48 h followed by 7 h after re-plating in the same conditions as in A (*P<0.05, **P<0.01, n=3).

Since miR-5096 was described as a pro-invasive factor [10], we investigated the motility of U87 cells using time-lapse microscopy. Figure 4A shows typical trajectories of U87 cells, either loaded with an empty vector or a miR-5096 inhibitor or a miR-5096 mimic or exposed to barium, with cell paths being plotted from a same initial position in an XY plan. Both empty vector and miR-5096 inhibitor loaded cell populations showed extended trajectories while miR-5096 mimic loaded and barium-treated cells showed random trajectories uniformly distributed on the plan, without preferential direction in cell motion. Further measurements showed that empty vector and miR-5096 inhibitor loaded cells demonstrated an increased and persistent cell migration, with a corresponding decrease in angle changes during migration (Figure 4B, 4D) and a similar velocity (Figure 4C). In contrast, miR-5096 mimic loaded and barium-treated cells showed significant less persistent migration, even though their ability to move remained similar to that of the two other groups (*i.e*. same velocity; Figure 4C). Cell imaging with a higher magnification revealed than empty vector-transfected cells developed single broad lamellae, also known as lamellipodia, at their leading edge (Figure 4E). In contrast, barium-treated and miR-5096 mimic loaded cells extended multiple finger-like projections also described as filopodia (Figure 4E, 4F). These changes are a likely explanation for the decrease in migratory persistence and the increase in angle changes during migration observed in barium-treated and miR-5096 mimic loaded cells.

### miR-5096 and Kir4.1 knockdown similarly increase glioma cell invasion

**Figure 5:**
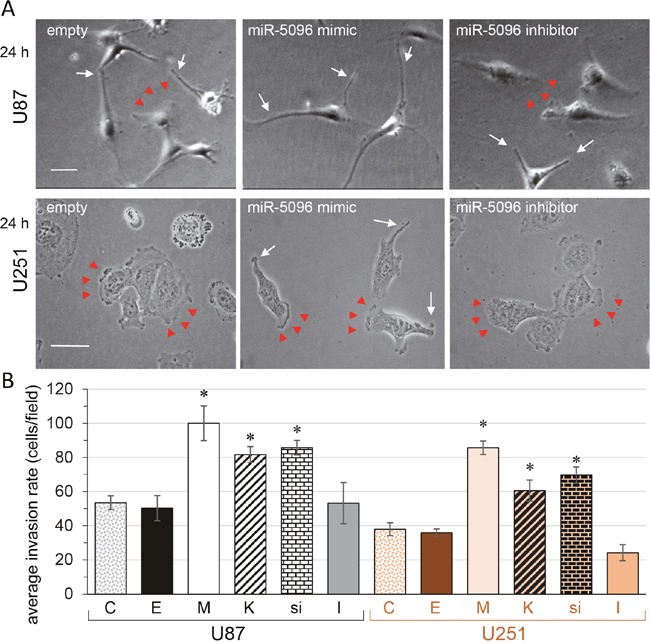
miR-5096, barium or silencing Kir4.1 expression, increase glioblastoma cell invasion **(A)** Images of individual cells showing the wound edge (lamellipodia; arrow heads) and finger-like projections (filopodia; white arrows) observed 24 h after re-plating (U87, upper panels; U251, lower panels; bar 50 μm). **(B)** Invasion assay. Boyden chamber with matrigel-precoated filter insert was used to measure *in vitro* invasiveness of transfected glioblastoma cells (U87, black; U251, orange). After 24 h of incubation, cells that migrated through the filter, were stained and counted. Columns represent the mean number of cells per field of at least six fields made in triplicate, in four independent experiments (mean ± SD; *P<0.05 *vs* empty for each group; *n* = 4).

24 h after re-plating, filopodia were also observed at the leading edge of empty vector- and miR inhibitor-transfected U87 cells (Figure 5A; upper panel). In contrast, U251 cells always showed well-organized lamellipodia (Figure 5A; lower panel). However, miR-mimic-transfected U251 changed cell shape somewhat from polygonal appearances to spindle forms with membrane ruffles and filopodia formation.

Given that there is a role for filopodia in invasion of cancer cells (*i.e*. invadopodia; [18, 28]), we examined the effect of Kir4.1 depletion, *i.e*. by miR-5096 loading, barium blockage or *si*RNA knockdown, on the invasion rate of the two cell lines by using Boyden chamber with matrigel-precoated filter inserts (Figure 5B). Over a 24-hour period, the invasiveness of U87 and U251 was increased by two-fold following Kir4.1 depletion. Note that inhibition of endogenous miR-5096 also decreased by about two-fold the invasion of U251 cells.

## DISCUSSION

The main finding of this study is that miR-5096 significantly decreases the expression of Kir4.1 channel protein in glioblastoma cells. Consequences include an increased release of extracellular vesicles, and an enhanced number of filopodia projections that alter cell motility. Because Kir4.1 channel protein is specifically expressed in glia cells, these effects are cell specific and cannot be detected in surrounding healthy endothelial cells to which this microRNA was shown to be transferred [16].

Overloading miR-5096 inhibits the inwardly rectifying, barium-sensitive K^+^ current in U87 cells. Although our study is focused on Kir4.1 channels, we cannot exclude that the reduction and/or blockage of these channels could be compensated by Cl- and water movements. Indeed K^+^ moves together with Cl^−^ and H_2_O in a Boyle-Conway type scenario. In support of such a scenario Kir4.1 and Aqp4 [29] and Cl^−^ channels [30] co-localized to astrocytic endfeet, forming a multi-protein dystrophin-glycoprotein complex [21]. In addition, the chloride intracellular channel 1 (CLIC1) is involved in development of the most aggressive human tumors, including glioblastoma [31–33]. Increased chloride conductivity could increase cell mobility (see [34]); indeed, the reverse potential of the current was depolarized in miR-5096 loaded cells (≈ -40 mV; i.e. near the equilibrium potential for chloride ions), suggesting the increase of an additional background component. This aspect is in progress. In addition to Kir4.1 channels, the inwardly rectifying two-pore domain K^+^ (K2P) channels, namely TWIK-1 (sensitive to barium) and TREK-1 (insensitive to barium), are known to be highly expressed in astrocytes [35, 36]. However, both TWIK-1 and TREK-1 proteins are located in the intracellular compartments and not in the plasma membrane [37, 38], excluding their contribution to the whole-cell currents. The genetic deletion of TREK-1 alone or together with TWIK-1 produced no obvious alteration in the basic electrophysiological properties of astrocytes [38]. Thus, it is unlikely that K2P channels substantially contribute to the current pattern of glioblastoma cells. Therefore Kir4.1 channels constitute the main outwardly rectifying current recorded in our patch-clamp conditions.

A decrease in functional Kir4.1 channels in glial cells has now been described in a variety of human neurological diseases [2]. The decrease in Kir4.1 expression observed in glioblastoma cells, may contribute to the impaired K^+^ buffering and increased propensity for seizures [8]. We show here that miR-5096 overexpression is in part responsible from this down regulation, thereby contributing to the aberrant growth of these glial derived tumor cells [8]. Such a regulation is reminiscent of what is observed in human corneal cells in which miR-205 stimulates wound healing by targeting the *KCNJ10* gene [39]. More generally, the expression of membrane ion channels is frequently modulated by microRNAs, e.g. the sodium channel is down-regulated by miR-30b in dorsal root ganglion [40], while the voltage-gated K^+^ channel Kv4.2 depends on miR-223-3p in cardiac myocytes [41].

Another consequence of a decrease in the expression of Kir4.1 channels is an altered cell behavior with increased formation of filopodia [42]. Neurite outgrowth in neuroblastoma cells similarly depends on K^+^ currents [43]. While the cell velocity is not affected by potassium exchange, cell migration is decreased in barium-treated and miR-5096 mimic loaded glioblastoma cells. A link has been previously established between α9β1 integrin-mediated cell migration and the Kir4.2 channel pathway in glioma cells [44–46], suggesting that Kir4.1 could also functionally cooperate with integrins to modulate glioma cell migration [47]. In our study, when Kir4.1 is downregulated by siRNA or cells are loaded with miR-5096 mimic, there is an associated increase in glioma cell invasion. Moreover, a slight but significant decrease in U251 cell invasion was induced by the miR-5096 inhibitor, suggesting a contribution of the endogenous miR5096 to this cell type behavior. Taken together our results point out the role of Kir4.1 in glioma cell invasiveness. Whereas the mechanism by which Kir4.1 modulates glioma invasion is not elucidated, we could hypothesize that decrease in Kir4.1 expression may favor the assembly of cytoskeletal proteins, in particular actin microfilaments, in filopodia at the leading edge of motile cells causing an increase in cell invasion [13]. Interestingly, Kir4.1 channels are located at the end feet of filopodia forming synapses or attached to blood vessels [1, 19, 48]. In perivascular regions, Kir4.1 channels co-localize with the water channel AQP4 [21]. However, AQP4 and Kir4.1 are differentially redistributed in glioblastomas, suggesting that the mechanisms of clustering of AQP4 and Kir4.1 are distinct at the glial endfeet membrane domains [7]. Here, we show that miR-5096 induces a slight increase in the expression of AQP4 while down-regulates Kir4.1 expression. Since the apposition of Kir4.1 channels and AQP4 might underlie the effective co-transport of K^+^ and water in glial cells, miR-5096 effects on Kir4.1 could compromise the buffering capacity of glial cells, leading to water influx and contributing to cytotoxic edema.

To promote tumor growth and invasion, glioblastoma cells subvert the surrounding microenvironment by influencing activity of neighboring healthy cells. We have previously reported that, two days after cell loading, miR-5096 down regulated the expression of Cx43 in U87, preventing the formation of heterocellular gap-junctions between U87 and HMEC. In spite of the absence of gap junctions, U87 cells were still able to transfer miR-5096 to HMEC. Here, we show that miR-5096 increases the release of exosomes that contain miR-5096. Although the mechanism of exosome-derived miR-5096 uptake and processing in recipient HMEC cells is unknown, extracellular vesicles could explain the transfer of miRs between the two cell types. Such an increase in exosome release was also induced by silencing Kir4.1 with siRNA but not by external barium, suggesting that blocking Kir4.1 membrane channels was not involved. Moreover, the simple presence of HMEC in co-culture was able to increase the ability of U87 to release extracellular vesicles in basally expressing miR-5096 conditions (i.e. empty-transfected cells).

Altogether, the present study suggests that Kir4.1 represents a potential therapeutic target in glioblastoma as in a wide variety of other neurological disorders [27]. Although other channel proteins could be regulated by miR-5096 [49], and other microRNAs could also influence the expression and function of these channels [39–41], the use of K^+^ channel openers appears as a potential therapeutic tool for neurologic disorders including glioblastomas.

## MATERIALS AND METHODS

### Cells

Human microvascular endothelial cells (HMEC; Lonza, Switzerland) and glioblastoma cells (U87-MG; ATCC HTB-14) were grown in DMEM plus 10% FCS (5% CO_2_; 37°C). Glioblastoma U251-MG were a gift of Pr. M. Mesnil (STIM laboratory, Poitiers). In co-culture conditions, transfected cells (one type) were washed and mixed with non-transfected cells (the other type) in a ratio of 1:1 (i.e. each cell type was seeded at half the seeding density of the homotypic cultures). Cells were incubated 48 h in FCS-free media before use.

### Reagents

Rabbit polyclonal anti-Kir4.1 (extracellular; APC-165) and anti-AQP4 (AQP-004) were purchased from Alomone labs (Jerusalem, Israel). Rabbit polyclonal anti-HSP90 was from ThermoFisher. Rabbit polyclonal anti-CD9 and mouse monoclonal anti-Tsg101, anti-Hsc70 and anti-β-actin were from Santa Cruz Biotech. GW4869 was purchased from Calbiochem (Merck Chimie SAS, Fontenay-sous-Bois). Other chemicals were from Sigma-Aldrich.

### Transfection

Human hsa-miR-5096 mimic (mirVana TM miRNA, 4464066-MC22429) and hsa-miR-5096 inhibitor (4464084-MH22429) were purchased from Ambion (Invitrogen; Life Technologies). Cells were transfected by lipofectamine RNAiMAX according to the manufacturer's protocol (Invitrogen). For some exosome analysis, HMEC were also transfected with human hsa-miR-145-5p mimic (mirVana TM miRNA, 4464066-MC11480) or hsa-miR-145-5p inhibitor (4464084-MH11480).

To downregulate Kir4.1, cells were transfected with human *Kcnj10 si*RNA SMARTpool (30 nM) purchased from Dharmacon, with target sequences: (1) GAUCCGAGUUGCCAAUAUG; (2) CAAGUUGGAGGAGUCAUUA; (3) GAGCUGGU GCUGAUCCUAA; (4) GCUACAAGCUUCUGCUCUU. Control *si*RNA was also from Dharmacon (ThermoFisher, Fr). Note that Kir4.1 knockdown could be observed 24 h after *si*RNA transfection, and reached maximum depletion at 72 h after transfection (not shown). Therefore, cells were miR-loaded, one day after *si*RNA transfection, then cultured 48 h to analyze the functional effects of Kir4.1 depletion.

### Patch-clamp experiments

Within 48 h after transfection, U87 cells were plated overnight at a lower density in petri dishes to avoid any cell-to-cell contact. The whole-cell patch-clamp experiments were carried out at room temperature, using a patch-clamp amplifier (Axoclamp 200A, Axon Instruments). Cells were incubated in a standard bath solution containing (in mM): 136 NaCl, 5 KCl, 1 MgCl_2_, 1.8 CaCl_2_, 0.3 NaH_2_PO_4_, 10 Glucose, and 10 HEPES (pH 7.4). Patch pipettes (of 3 to 5 M≈) were filled with a solution containing (in mM): 20 KCl, 110 K-Aspartate, 1 MgCl_2_, 10 HEPES, 5 EGTA, 0.1 GTP, 5 Na_2_Phosphocreatinin, and 5 Mg-ATP (pH 7.2). Note that the addition of 5 mM EGTA and Mg^2+^-ATP into the pipette prevented the activation of Ca^2+^-activated or ATP-sensitive K^+^ currents. Voltage ramps were applied from -130 to 0 mV for 2 s (Holding Potential -60 mV), excluding the contribution of voltage-activated components to the recorded current. The amplitudes of membrane currents were normalized to the cell membrane capacitance and presented as picoAmpere/picoFarad (pA/pF). The Kir current was determined by the addition of 500 μM BaCl_2_ in the bath solution. Note that concentrations of more than 200-500 μM BaCl_2_ are conventionally used to block the purely Kir4.1-composed inwardly rectifying K^+^ current [18, 50, 51].

### Cell proliferation

CellTrace^TM^ Violet was used for tracking proliferation in glioblastoma cell cultures, by fluorescent dye dilution and flow cytometry, according to the manufacturer's protocol (Molecular Probes, ThermoFisher, Fr).

### Exosomes purification

After 48h of culture in FCS-free conditions, cell supernatants were collected as previously described [23]. Supernatants were sequentially centrifuged at 300 g for 10 min (4°C) then at 2,000 g for 10 min to remove cell debris. Exosomes were collected by ultracentrifugation at 100,000 g for 90 min then washed in filtered PBS. Size and concentration of exosomes were characterized using Nanosight LM10 (Amesbury, UK) and analyzed with NTA 3.1 software (Malvern). Concentrations were adjusted to the same number of cells (i.e. corresponding to the secretion from 4 × 10^6^ cells).

### Immunoblotting

Briefly, cells were lysed in RIPA buffer, and Western blots were performed with antibodies, as previously described [16]. Exosomes pellets were lysed in RIPA buffer containing protease inhibitor cocktail (Roche, Indianapolis, IN), then sonicated for 10 s. Insoluble material was pelleted by centrifugation for 15 min at 14,000 g at 4°C.

### RNA isolation and real-time PCR analysis

Total RNA was isolated from exosomes of homotypic cultures using TRIzol reagent (Invitrogen). Expression of miR-5096 (U87 exosomes) or miR-145 (HMEC exosomes) were determined using TaqMan miRNA assay (Invitrogen) according the manufacturer's protocols. Level of U6 snRNA (Ambion, 4427975-001973) was used as internal control for analysis.

First-strand DNA synthesis and semi-quantitative reverse transcription-polymerase chain reaction (RT-qPCR) were performed as described previously [51]. The relative expression level of *KCNJ10* (Kir4.1) was firstly examined by using the primer sequences: Kir4.1_F (GGAGGAGATCCTCTGGGGTT); Kir4.1_R (CCACTGGGAGATGCCACTTT); GAPDH_F (AAGC TCACTGGCATGGCC); GAPDH_R (GCCTGCTTCA CCACCTTC). RT-qPCR analyses were secondly performed, as described previously [52], using the following primers: Kir4.1_F (TATCAGAGCAGCCA CTTCACCTTC); Kir4.1_R (GGATCGTCTCGGCCC TCTTCTTAG) (GenBank accession number: NM_020269).

### Human HSP90α immunoassay

The soluble phase of cell supernatants was collected from the first ultracentrifugation used for exosome isolation. The quantitative detection of soluble HSP90α released by cells (4 × 10^6^ cells) was made by ELISA (ADI-EKS-895; Enzo Life Sci.; Switzerland), according to the manufacturer's instructions.

### Cell dynamics analysis by time lapse microscopy

After 48h of culture in FCS-free conditions, transfected U87 cells were trypsinized and suspended in 6-well plates (30,000 cells/well) in DMEM containing 0.5%-FCS (at 37°C with 5% CO_2_). Time-lapse video microscopy was performed by Olympus IX-81-ZDC. Images were taken every 3 min for 15 h using Andor Iq2.0 software, then were analyzed using Imaris software (BitPlane). In each well, 40 single cells were tracked for analysis and each well analysis was triplicated. For short term assays (after 7 hours of incubation at 37°C), 80 single U87 cells were scored for the number of processes per cell.

### Cell invasion assay

Invasive properties of GBM cell lines were explored using a Boyden chamber containing 8 μm inserts that are pre-coated on the top with ECM matrix gel (basement membrane), in 24-well tissue culture plates (Cell Biolabs Inc., Euromedex, Fr.). Two days after transfection with *si*RNA and/or miR loading, cells were seeded at 40,000 cells/well and allowed to migrate for 24 h in FCS-free culture conditions. The bottom chamber was filled with DMEM containing 10% FCS as a chemoattractant. Non-invading cells were removed by wiping the upper surface of the insert with a cotton swab. Invasive cells on the bottom of the insert were stained and quantified in at least six random fields per filter. Experiments were repeated three times in triplicate.

### Statistical analysis

Results are expressed as mean ± SD. A Mann-Whitney *U* test was used to compare data groups. Statistics were also made with Tanagra software using a Kruskal-Wallis 1-way ANOVA. In all cases, *P values < 0.05 were significant.

## SUPPLEMENTARY MATERIALS FIGURES


