# Supplementary material for: Modulation of the inwardly rectifying potassium channel Kir4.1 by the pro-invasive miR-5096 in glioblastoma cells
Source: Oncotarget. 2017 Apr 7;8(23):37681–93. doi: 10.18632/oncotarget.16949 (PMC5514940; doi:10.18632/oncotarget.16949)
Supplement: Supplementary file 1 [file oncotarget-08-37681-s001.pdf]

Modulation of the inwardly rectifying potassium channel Kir4.1 by the pro-invasive miR-5096 in glioblastoma cells

SUPPLEMENTARY MATERIALS

SUPPLEMENTARY FIGURE

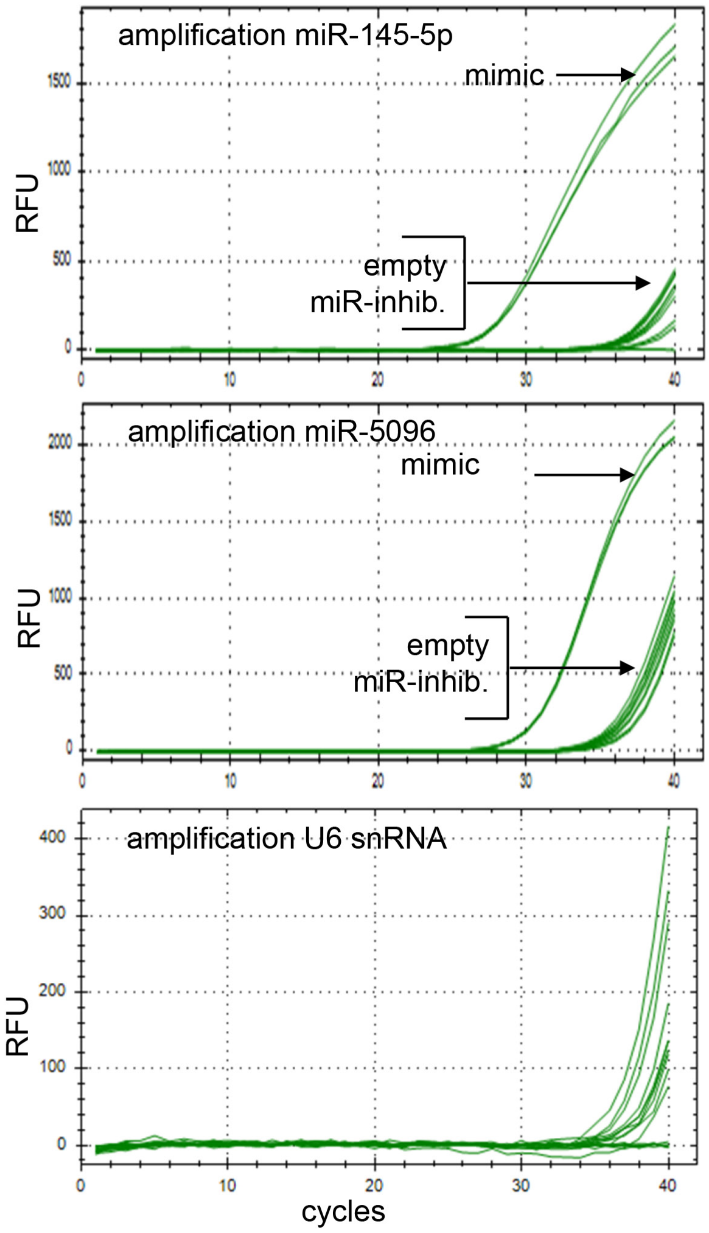

Supplementary Figure 1: miR-145-5p and miR-5096 are detected in exosomes released from microvascular endothelial cells (HMEC) and glioblastoma cell line (U87), respectively. Cells were transfected empty, miR-inhibitor (30 nM) or miR-mimic (30 nM), then cultured separately for 48h. Exosomes were collected from supernatants of homotypic cultures of HMEC (miR-145) and U87 (miR-5096). The miR contain was amplified by real-time qPCR (representative amplification curve made in triplicate). Note that U6 snRNA (used an intracellular control) was not detected in exosomes. Thus, levels of miR-145 or -5096 could not be quantified relative to levels of U6 snRNA, as usually made.
